# Cardiovascular risk factors in autoimmune thyroiditis: role of adiponectin, resistin, inflammation and auto-immunity - a cross-sectional study

**DOI:** 10.3389/fendo.2026.1865915

**Published:** 2026-10-05

**Authors:** Celestino Neves, João Sérgio Neves, Oksana Sokhatska, Luís Delgado

**Affiliations:** 1 Department of Endocrinology, Diabetes and Metabolism, Unidade Local de Saúde São João, Porto, Portugal; 2 Faculty of Medicine of University of Porto, Porto, Portugal; 3 RISE-Health, Faculty of Medicine, University of Porto, Porto, Portugal; 4 Basic and Clinical Immunology, Department of Pathology, Faculty of Medicine, University of Porto, Porto, Portugal; 5 Laboratory of Immunology, Department of Clinical Pathology, Unidade Local de Saúde São João, Porto, Portugal

**Keywords:** adipokines, autoimmune thyroiditis, cardiovascular risk factors, lipid profile abnormalities, thyroid function

## Abstract

**Objective:**

Autoimmune thyroiditis (AIT) has been associated with an increased cardiovascular risk. Whether adipokines, inflammation and auto-immunity contribute to this increased risk is uncertain. Our aim was to evaluate the interrelationships of adipokines, inflammation, auto-immunity, and thyroid function with cardiovascular risk in autoimmune thyroiditis.

**Methods:**

Sixty-two patients with AIT, with normal free T4 (FT4) and free T3 (FT3), without other comorbidities were studied. Patients were divided into two groups: AIT with thyroid-stimulating hormone (TSH) ≤2.5 mU/L and AIT with TSH >2.5 mU/L. Parameters of thyroid function tests, serum lipid, high sensitivity C-reactive protein (hs-CRP) and homocysteine levels, anti-thyroglobulin antibodies, anti-thyroid peroxidase (TPO) antibodies, adiponectin and resistin were measured.

**Results:**

Patients with TSH >2.5 mU/L had significantly higher body mass index, higher low density lipoprotein (LDL)-cholesterol levels, and lower lipoprotein(a) levels. No differences in triglycerides, apolipoprotein A1 (ApoA1), apolipoprotein B (ApoB), adiponectin, or resistin levels were observed between groups. In the overall cohort, adiponectin levels were positively correlated with high density lipoprotein (HDL)-cholesterol and lipoprotein(a), whereas resistin was positively correlated with hs-CRP and triglyceride levels. Hs-CRP levels were positively correlated with anti-thyroglobulin antibody titers, and homocysteine levels were negatively correlated with FT3. In the subgroup with TSH >2.5 mU/L, FT4 was positively correlated with ApoA1 and HDL-cholesterol and negatively correlated with lipoprotein(a), while resistin was positively correlated with TSH. In patients with TSH ≤2.5 mU/L, adiponectin levels were negatively correlated with TSH.

**Conclusion:**

Our study highlights a complex interrelationship between cardiovascular risk factors in patients with AIT. Patients with AIT and TSH >2.5 mU/L may have a more atherogenic lipid profile.

## 1 Introduction

Autoimmune thyroiditis (AIT) is one of the most commonly diagnosed organ-specific auto-immune disorders (1, 2). The pathological hallmarks of AIT are the production of thyroid-specific auto-antibodies in parallel to an inflammatory infiltration of the organ by T cells, with thyrocyte apoptosis resulting in a progressive organ failure and hypothyroidism (3, 4). The spectrum of thyroid auto-immune dysfunction is associated with different levels of cardiovascular risk, as demonstrated by independent changes in the severity of risk factors that have been linked to cardiovascular morbidity in many studies (5–7). The increased cardiovascular risk seen in thyroid dysfunction is multifactorial and probably related to lipid profile changes, endothelial dysfunction, coagulation disturbances and also metabolic, hormonal and hemodynamic changes (8, 9). The classic lipid profile changes in overt hypothyroidism are raised very low-density lipoproteins (VLDL), low-density lipoproteins (LDL) and apoB, with a correlation of total cholesterol and LDL cholesterol with thyroid-stimulating hormone (TSH) (9). Subclinical hypothyroidism, with thyroid hormones within the reference range but with elevated TSH, can also be associated with an unfavorable lipid profile, although with no consistent results in different studies (10–12).

Thyroid hormones are important regulators of intermediate and energy metabolism (13, 14), namely of fat stocks and adipose tissue. Previous studies have suggested a significant influence of thyroid hormones on the regulation of human adipokines; however, the results published to date have been inconsistent between studies (15–19).

Adipokines, secreted by several components of adipose tissue, play an important role in glucose, lipid and lipoprotein metabolism, as well as in coagulation and inflammatory processes, thus modulating risk factors for atherosclerosis (20). Circulating adiponectin levels are inversely correlated with various cardiovascular risk factors, such as LDL cholesterol and triglyceride levels (21) and seem to have an anti-atherogenic role. Resistin, another adipokine (22, 23), has been shown in human studies to be positively associated with leukocytes, C-reactive protein (CRP) and IL6 (22).

Among those with autoimmune thyroiditis a TSH cutoff point of 2.5 mU/L has been proposed as better limit, compared to upper limit of the normal range, to define the risk of cardiometabolic complications (24, 25). A TSH >2.5 mU/L has been associated with adverse cardiometabolic features in independent populations, including metabolic syndrome (26), increased visceral adiposity and dyslipidemia (24), and increased all-cause mortality (25).

In this study we aimed to evaluate the interrelationships of adipokines, inflammation and thyroid function with cardiovascular risk in AIT.

## 2 Methods

Sixty-two patients with AIT, aged 18 years or older, followed-up in outpatient department of São João Hospital (Porto, Portugal) between 2010 and 2020 were enrolled in this study. These patients were recently diagnosed by a positive result in anti-peroxidase (anti-TPO) and anti-thyroglobulin (anti-Tg) serum antibodies test, and diffuse heterogeneous hypoechogenic ultrasound pattern. Two groups were considered: patients with TSH ≤2.5 mU/L and patients with TSH >2.5 mU/L. The 2.5 mU/L cut-off was chosen because, within the conventional euthyroid range, a TSH above this threshold has been repeatedly associated with adverse cardiometabolic features and with a higher rate of progression to overt hypothyroidism in TPO-positive subjects (24–26, 28, 29).

Only patients with stable TSH values (defined as having two thyroid hormone profiles in a 3-month span with TSH within the normal range) were included. Patients treated with thyroid hormones were not included in this study. Participants with free triiodothyronine (FT3) or free thyroxin (FT4) levels outside of the normal range were excluded (FT3 reference range: 1.71-3.71 pg/mL, FT4 reference range: 0.70-1.48 ng/dL).

Exclusion criteria, in addition to hypothyroidism and prior thyroid disease, were pregnancy, smoking, hepatic or renal dysfunction, diabetes mellitus, coronary heart disease, arrhythmias, stroke, chronic inflammatory disease, infectious disease or malignant disease. Patients receiving lipid-lowering, antihypertensive or anti-inflammatory drugs were not included. Patients gave their informed consent to participate. The study was approved by the Ethics Committee of São João University Hospital Centre (number 197/18, approved on 17/10/2018) and conducted in accordance with the Declaration of Helsinki.

### 2.1 Laboratorial methods

Serum TSH, FT3, FT4 and homocysteine were measured using Abbott Diagnostics Architect i2000 automatic immunochemistry system. ApoA1, ApoB, and Lipoprotein(a) [Lp(a)] were measured by an immunoturbidimetric assay, and total cholesterol, high density lipoprotein (HDL-C), LDL-cholesterol and triglycerides by a colorimetric assay, using the Olympus Diagnostics AU5400 analyzer system (Beckman Coulter, USA). High sensitivity-CRP (hs-CRP) levels were measured by nephelometric latex high sensitivity assay BN II™ System (Siemens Healthcare Diagnostics). Anti-Tg and anti-TPO antibodies were measured in the ADVIA Centaur^®^ Immunoassay system (Siemens Healthcare Diagnostics). In accordance with the manufacturer’s recommended thresholds, antibody positivity was defined as anti-TPO >5.6 IU/mL and anti-Tg >4.1 IU/mL.

Adiponectin and resistin were measured by a multiplex analysis assay (Millipore, Hamburg, Germany). Briefly, the appropriate cytokine standards, controls and plasma samples were diluted in assay buffer and added to pre-wet filter plates. Fluorescent beads containing adiponectin and resistin antibodies were added and incubated on a plate shaker overnight, at 4°C. The wells were washed, and the samples were incubated with detection antibodies, for 30 minutes at room temperature and with streptavidin-phycoerythrin, under the same conditions. After washing, beads were resuspended with sheath fluid and analyzed on a Luminex 200™. Mean fluorescent intensity data were analyzed using the Luminex 100 Integrated System version 2.3 (Luminex Corporation, USA).

### 2.2 Statistical methods

Data are expressed as mean ± standard deviation for normally distributed variables and as median (P25–75) for non-normally distributed variables. Comparison between groups by the Student t-test and Mann-Whitney U-test were used. Spearman’s rank correlation test was used to evaluate univariate relationships. All statistical analyses were done using SPSS for Windows (SPSS Inc., Chicago, IL). A two-tailed p value <0.05 was considered statistically significant.

## 3 Results

### 3.1 Participant characteristics

The study population comprised 62 patients with autoimmune thyroiditis, with a mean age of 47.7 ± 16.7 years; 58 participants (94%) were women. The mean body mass index (BMI) was 28.6 ± 8.0 kg/m². Patients were stratified according to serum TSH levels into two groups: TSH >2.5 mU/L (n=33) and TSH ≤2.5 mU/L (n=29).

The two groups were similar in age and sex distribution. However, patients with TSH >2.5 mU/L had a significantly higher BMI than those with TSH ≤2.5 mU/L. Although FT3 concentrations remained within the reference range in both groups, they were significantly lower in patients with TSH >2.5 mU/L, whereas FT4 levels did not differ significantly between groups. Anti-TPO antibody titers were significantly higher in the TSH >2.5 mU/L group, while anti-thyroglobulin antibody levels showed no statistically significant difference (p=0.06) (**Table 1**).

**Table 1:** Demographic characteristics, thyroid function and thyroid antibodies of the study population.

Characteristic	TSH >2.5 mU/L	TSH ≤2.5 mU/L	
	(n=33)	(n=29)	P-value
Age (years)	46 ± 17.5	49 ± 16	0.47
Sex (male/female)	1/32	3/26	0.52
BMI (kg/m^2^)	30.5 ± 9.6	26.4 ± 5.1	0.04
TSH (mU/L)	3.55 (2.98-5.57)	1.21 (0.74-1.84)	<0.001
Free T3 (pg/mL)	2.56 (2.34-2.89)	2.74 (2.56-2.93)	0.03
Free T4 (ng/dL)	1.01 (0.83-1.10)	1.04 (0.91-1.16)	0.34
Anti-Tg (IU/mL)	107.7 ± 120.3	77.6 ± 106.5	0.06
Anti-TPO (IU/mL)	844.9 ± 562.5	427.3 ± 535.0	<0.001

Anti-Tg, anti-thyroglobulin antibody; anti-TPO, anti-thyroid peroxidase antibody; BMI, body mass index.

### 3.2 Lipid profile

Patients with TSH >2.5 mU/L had a less favorable lipid profile. LDL-cholesterol levels were significantly higher in this group compared with those with TSH ≤2.5 mU/L. Total cholesterol also tended to be higher, although the difference did not reach statistical significance (p=0.07).

No significant difference between groups was observed for HDL-cholesterol, triglycerides, apoA1, or apoB. In contrast, lipoprotein(a) concentrations were significantly lower in the TSH >2.5 mU/L group (**Table 2**).

**Table 2:** Lipid profile, hs-CRP, homocysteine and adipokines of the study population.

Parameter	TSH >2.5 mU/L	TSH ≤2.5 mU/L	
	(n=33)	(n=29)	P-value
Total-cholesterol, mg/dL	221.3 ± 50.7	199.1 ± 39.3	0.07
HDL-cholesterol, mg/dL	56.1 ± 15.6	58.3 ± 16.3	0.60
LDL-cholesterol, mg/dL	137.7 ± 33.4	122.5 ± 27.4	0.02
Triglycerides, mg/dL	121.7 ± 77.4	113.5 ± 57.1	0.65
Lipoprotein (a), mg/dL	21.8 ± 28.2	46.1 ± 39.9	0.03
Apolipoprotein A1, mg/dL	143.3 ± 20.0	138.9 ± 21.8	0.49
Apolipoprotein B, mg/dL	106.6 ± 32.9	94.8 ± 17.1	0.18
hs-CRP, mg/dL	0.57 ± 0.57	0.31 ± 0.25	0.06
Homocysteine, µmol/L	12.69 ± 20.90	9.22 ± 5.34	0.83
Adiponectin, µg/mL	20.3 ± 9.4	20.2 ± 13.2	0.61
Resistin, ng/mL	13.9 ± 14.0	19.9 ± 22.7	0.17

hs-CRP, high-sensitivity C-reactive protein.

### 3.3 Hs-CRP levels and homocysteine

Hs-CRP levels tended to be higher in patients with TSH >2.5 mU/L, although this difference was not statistically significant. Homocysteine levels were similar in the two groups (**Table 2**).

### 3.4 Adiponectin and resistin

Adiponectin and resistin levels did not differ significantly between the two TSH groups. Adiponectin concentrations were 20.3 ± 9.4 µg/mL in patients with TSH >2.5 mU/L and 20.2 ± 13.2 µg/mL in those with TSH ≤2.5 mU/L (p=0.61). Resistin levels were also comparable between groups (13.9 ± 14.0 vs 19.9 ± 22.7 ng/mL, p=0.17) (**Table 2**).

### 3.5 Correlations in the overall study population

In the overall cohort, adiponectin levels were positively correlated with HDL-cholesterol (r=0.30, p=0.02) and lipoprotein(a) (r=0.32, p=0.04). Resistin levels were positively correlated with triglycerides (r=0.27, p=0.04) and hs-CRP (r=0.34, p=0.01). In addition, hs-CRP levels were positively correlated with anti-Tg antibody titers (r=0.33, p=0.01), whereas homocysteine levels were negatively correlated with FT3 (r=-0.36, p=0.005) (**Figure 1**).

**Figure 1:**
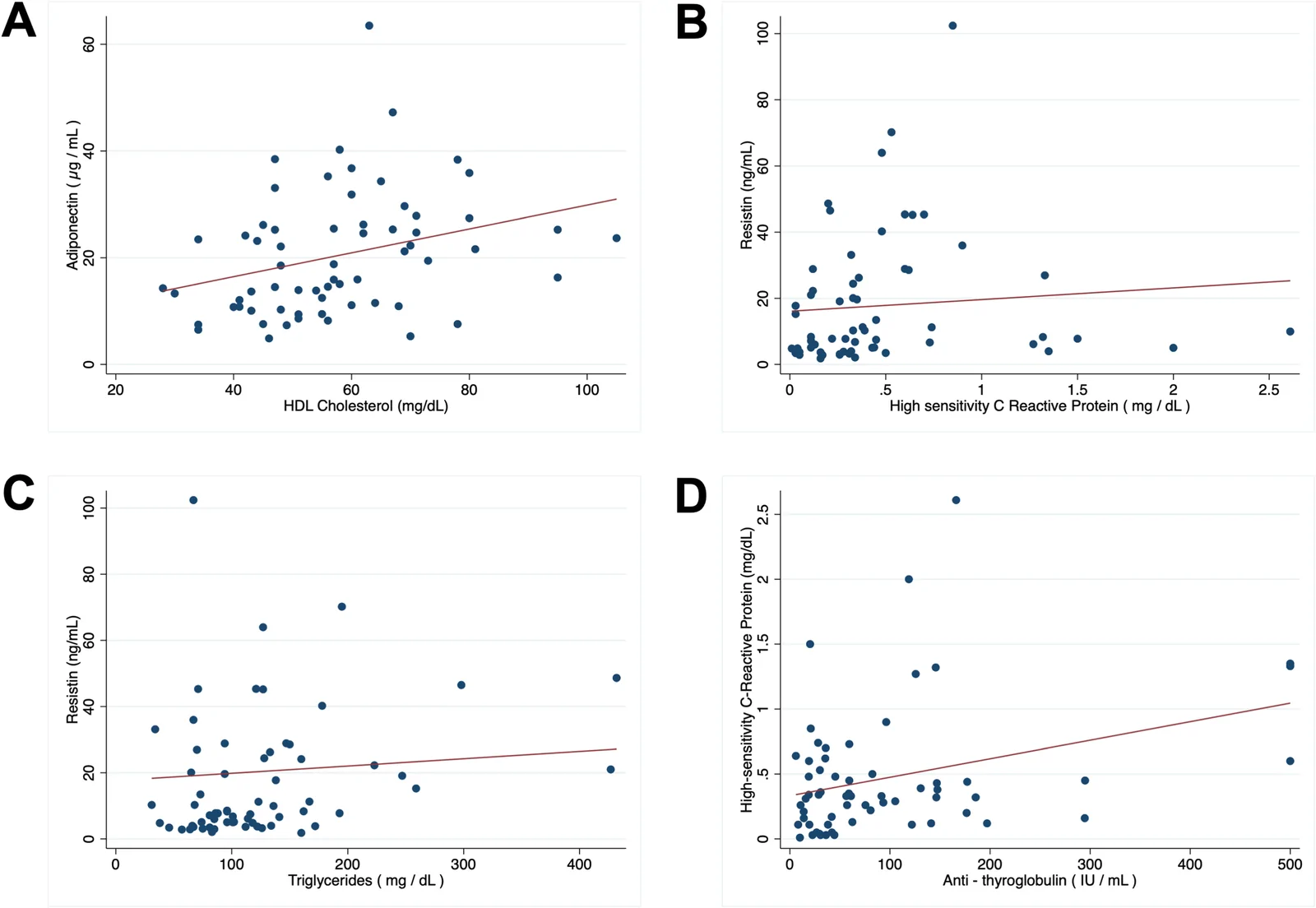
Correlations in the overall study population. **(A)** Correlation of adiponectin with HDL-cholesterol (r=0.30, p=0.02); **(B)** Correlation of resistin with hs-CRP (r=0.34, p=0.01); **(C)** Correlation of resistin with triglycerides (r=0.27, p=0.04); **(D)** Correlation of hs-CRP with anti-Tg antibody titers (r=0.33, p=0.01).

### 3.6 Correlations according to TSH subgroup

In patients with TSH >2.5 mU/L, FT4 was positively correlated with apoA1 (r=0.41, p=0.04) and HDL-cholesterol (r=0.48, p=0.01), and negatively correlated with lipoprotein(a) levels (r=-0.44, p=0.03). In this subgroup, resistin was positively correlated with TSH (r=0.38, p=0.03). In patients with TSH ≤2.5 mU/L, adiponectin levels were negatively correlated with TSH (r=-0.57, p=0.001).

## 4 Discussion

In this study of euthyroid patients with autoimmune thyroiditis (AIT), we found that individuals with TSH levels >2.5 mU/L exhibited a less favorable cardiometabolic profile, characterized by higher LDL-cholesterol concentrations and a trend toward higher hs-CRP levels, despite thyroid hormone concentrations remaining within the reference range. These findings support the concept that subtle variations in thyroid function within the euthyroid range may influence cardiovascular risk markers in patients with thyroid autoimmunity.

Our observation of higher LDL-cholesterol in patients with TSH >2.5 mU/L is consistent with previous reports suggesting that even normal-high TSH may adversely affect lipid metabolism before overt hypothyroidism develops. Several studies have demonstrated a positive association between TSH and total/LDL cholesterol within the reference range, particularly in individuals with thyroid autoimmunity or subclinical hypothyroidism (10–12). Thyroid hormones regulate LDL receptor expression and hepatic cholesterol metabolism, and small reductions in thyroid hormone bioactivity may impair LDL clearance even when FT4 and FT3 remain normal (9, 27). The positive correlations observed between FT4 and both HDL and apoA1 in the subgroup with TSH >2.5 mU/L further reinforce the biological relevance of subtle thyroid hormone variations in lipid homeostasis.

The higher anti-TPO antibody titers in the TSH >2.5 mU/L subgroup suggest that greater autoimmune activity may contribute to progressive thyroid dysfunction and metabolic impairment. This is in agreement with longitudinal studies showing that anti-TPO positivity predicts progression toward hypothyroidism and may identify individuals with more active autoimmune-mediated thyroid destruction (28, 29). Whether thyroid autoimmunity per se contributes independently to cardiovascular risk remains debated; however, autoimmune-mediated inflammation has been proposed as an additional mechanism linking AIT to endothelial dysfunction and atherosclerosis beyond thyroid hormone alterations alone (30).

Although hs-CRP levels did not differ significantly between groups, the near-significant trend toward higher hs-CRP in patients with TSH >2.5 mU/L may indicate a low-grade inflammatory milieu associated with mild thyroid failure or more active autoimmunity. Previous studies have reported increased inflammatory markers in both overt and subclinical hypothyroidism, potentially contributing to endothelial dysfunction and cardiovascular risk (31, 32). The lack of statistical significance in our cohort may be related to limited sample size.

Regarding adipokines, we found no significant differences in adiponectin or resistin levels between TSH-defined groups, suggesting that mild differences in thyroid function within the euthyroid range may not substantially influence adipokine concentrations in AIT. This aligns with some previous reports showing no clear association between thyroid status and adiponectin or resistin (17, 33), although the literature remains inconsistent (34). Importantly, adiponectin correlated positively with HDL-cholesterol, supporting its known anti-atherogenic role, while resistin correlated positively with hs-CRP and triglycerides, consistent with its proposed pro-inflammatory and pro-atherogenic effects (20–23).

A novel finding of our study was the positive correlation between TSH and resistin in patients with TSH >2.5 mU/L. This may suggest that normal-high TSH is associated with enhanced pro-inflammatory adipokine signaling in AIT. Experimental and clinical studies have proposed that resistin may link thyroid dysfunction, inflammation, and insulin resistance (35), although data remain limited and inconsistent. The absence of corresponding differences in absolute resistin concentrations between groups suggests that this relationship may be subtle and detectable only when TSH is above 2.5 mU/L.

Interestingly, Lp(a) concentrations were lower in the higher TSH subgroup, an unexpected finding given the generally adverse lipid profile associated with hypothyroidism. Prior literature regarding thyroid function and Lp(a) is conflicting, with studies reporting increased, unchanged, or decreased Lp(a) levels in hypothyroidism (36, 37). This inconsistency likely reflects the strong genetic determination of Lp(a) and its limited responsiveness to metabolic factors.

Taken together, our findings support the hypothesis that a TSH threshold of 2.5 mU/L may identify a subgroup of euthyroid AIT patients with early metabolic alterations associated with increased cardiovascular risk. This is in line with prior studies suggesting that cardiometabolic risk may increase at TSH levels below the conventional upper limit of normal in susceptible populations (24, 26). Nevertheless, whether this threshold should influence clinical management remains uncertain and requires prospective outcome-based validation.

This study has limitations that should be acknowledged. First, its cross-sectional design precludes causal inference regarding the relationships observed. Second, the relatively small sample size may have limited statistical power, particularly for inflammatory and adipokine between-group comparisons, and reduces the external generalizability of our findings; subgroup analyses by TSH stratum are particularly susceptible to type II error and should be regarded as exploratory and hypothesis-generating. Third, BMI was higher in the TSH >2.5 mU/L subgroup, which may have partially confounded metabolic and adipokine findings despite similar obesity prevalence categories. Fourth, our biomarker panel was focused on three mechanistically complementary mediators — hs-CRP (low-grade inflammation), adiponectin (anti-atherogenic adipokine) and resistin (pro-atherogenic adipokine) — together with a comprehensive conventional lipid profile and homocysteine. While this panel captures the principal pathways implicated in thyroid-related cardiovascular risk, it does not constitute a predictive model of cardiovascular events. Broader profiling (e.g. IL-6, TNF-α, leptin, visfatin, PAI-1, oxidized LDL) and integration with subclinical vascular outcomes (carotid intima-media thickness, flow-mediated dilation) will be necessary in future studies to translate the mechanistic associations described here into improved cardiovascular risk stratification in AIT. Finally, we did not include a non-AIT control group, preventing assessment of whether the observed associations are specific to thyroid autoimmunity.

In conclusion, euthyroid patients with autoimmune thyroiditis and TSH >2.5 mU/L demonstrate subtle but potentially relevant adverse changes in cardiovascular risk markers, particularly LDL-cholesterol, together with associations between thyroid function and resistin. These findings suggest that normal-high TSH within the euthyroid range may have metabolic significance in AIT and support further investigation into the clinical relevance of lower TSH thresholds in this population.

## Data Availability

The datasets generated for this study are available from the corresponding author upon reasonable request. Requests to access the datasets should be directed to Celestino Neves, mcelestinoneves@gmail.com.
